# Assessing past versus present severe acute respiratory coronavirus virus 2 (SARS-CoV-2) infection: A survey of criteria for discontinuing precautions in asymptomatic patients testing positive on admission

**DOI:** 10.1017/ice.2023.147

**Published:** 2024-02

**Authors:** Shruti K. Gohil, Annabelle De St. Maurice, Deborah S. Yokoe, Stuart H. Cohen, Francesca J. Torriani, Jonathan D. Grein, Philip A. Robinson, Shannon Mabalot, Jessica Park, Paula Pedrani, Richard Platt, Susan S. Huang

**Affiliations:** 1 Epidemiology & Infection Prevention Program, University of California, Irvine Health (UC Irvine Health), Irvine, California; 2 Division of Infectious Diseases, University of California, Irvine School of Medicine, Irvine, California; 3 Division of Pediatric Infectious Diseases, David Geffen School of Medicine at University of California Los Angeles, Los Angeles, California; 4 University of California, San Francisco, Division of Infectious Diseases, San Francisco, California; 5 University of California, Davis, Division of Infectious Diseases, Davis, California; 6 University of California, San Diego, Division of Infectious Diseases, San Diego, California; 7 Cedars Sinai Medical Center, Division of Infectious Diseases, Los Angeles, California; 8 Hoag Memorial Hospital Presbyterian, Department of Infection Prevention, Newport Beach and Irvine, California; 9 Infection Prevention, Sharp Metropolitan Medical Campus, San Diego, California; 10 Department of Population Medicine, Harvard Pilgrim Healthcare Institute, Harvard Medical School, Boston, Massachusetts

## Abstract

Infection prevention program leaders report frequent use of criteria to distinguish recently recovered coronavirus disease 2019 (COVID-19) cases from actively infectious cases when incidentally positive asymptomatic patients were identified on routine severe acute respiratory coronavirus virus 2 (SARS-CoV-2) polymerase chain reaction (PCR) testing. Guidance on appropriate interpretation of high-sensitivity molecular tests can prevent harm from unnecessary precautions that delay admission and impede medical care.

The coronavirus disease 2019 (COVID-19) pandemic has led to policies and practices to prevent the spread of severe acute respiratory coronavirus virus 2 (SARS-CoV-2) in healthcare settings where contagious patients come to receive medical care.^1^ Universal admission and preprocedural testing for SARS-CoV-2 is commonly performed regardless of symptoms to ensure rapid application of COVID-19 precautions for patients who may be experiencing minimal symptoms or may be presymptomatic because patients are potentially contagious 2 days prior to symptom onset.^2^

COVID-19 polymerase chain reaction (PCR) tests are commonly preferred because of their greater sensitivity compared to antigen tests. However, PCR-positivity can persist for 3–5 months, long after contagiousness is over.^3–5^ This finding has led to unnecessary concern and precautions for contagiousness and unintended consequences, including delays in procedures or appropriate placement (eg, waiting for a single room, refusals for transfer to rehabilitation or skilled nursing facilities), restricted visitation, or compromised medical care (eg, admission to medical instead of psychiatric unit).^6–9^

## Methods

From March to April 2021, we conducted a structured survey of infection prevention program leaders who are members of (1) Society for Healthcare Epidemiology of America (SHEA) Research Network, (2) Centers for Disease Control and Prevention Epicenters, (3) University of California Health, and (4) California Healthcare-Associated Infections Metrics Group. The 14-question survey presented a series of hypothetical asymptomatic COVID-19 PCR-positive case scenarios that could be incidentally found on preprocedural or admission testing. The survey polled respondents on (1) whether they would consider the case recovered and not infectious, (2) whether they have cleared precautions in such cases, and if so, (3) how many transmission events occurred after discontinuing precautions. Case scenarios inquired about whether any solo or dual combination of 5 criteria were sufficient to determine clearance: (1) recent history of COVID-19 symptoms, (2) recent history of a household member with COVID-19, (3) SARS-CoV-2 test with high PCR cycle threshold (Ct), (4) 2 SARS-CoV-2 tests with high PCR Ct on separate days, and (5) IgG serology to SARS-CoV-2.

To create case scenarios, 1 month was used to define recent COVID-19 symptoms, 5 weeks was used for a recent household member with COVID-19, and a Ct >35 was used to indicate a high value. Notably, since all case scenarios were asymptomatic, the alternative value used for Ct was 30. The PCR Ct indicates the number of genomic amplification cycles required to detect SARS-CoV-2 (live or dead). Thus, lower Ct values indicate a higher burden of viral genetic material that is detected with fewer amplification cycles, and a high Ct value indicates a low burden (requiring several amplification cycles before detection).^10^ The survey instrument is provided in online Supplementary materials.

For each scenario, we aggregated responses (percentage of respondents) to the 3 questions about whether the case represented convalescent COVID-19, whether the respondent would recommend clearing precautions, and whether any known transmission events had occurred if the criteria had been used at their hospital. This study was exempt from approval by the University of California Irvine Institutional Review Board.

## Results

Respondents included 60 leaders of infection prevention programs among 117 hospitals (response rate 51%), including 58 (97%) in 25 US states. Among respondents, 56 (93%) were physicians and 51 (85%) were designated hospital epidemiologists. Experience in infection prevention was high, with 55 (92%) having at least 5 years of infection prevention experience, and 46 (77%) having at least 10 years of experience. Of represented hospitals, 46 (77%) were academic, 14 (23%) had 400–599 beds, and 28 (47%) had 600+ beds. At the time of the survey, 52 (87%) respondents were from hospitals that had cared for >200 COVID-19 patients and 46 (77%) were from hospitals that had cared for >600 COVID-19 patients.

Using solo criterion in an asymptomatic individual with incidentally discovered PCR positivity (Table 1), at least one-third would determine that person to be noninfectious and recovered from COVID-19 if they had a clear history of either recent COVID-like symptoms or a household contact with known COVID-19. The majority of those with that opinion have cleared COVID-19 precautions using these criteria without resultant COVID-19 transmission events. More than half of respondents would clear precautions if 2 PCRs had Ct >35 or if positive for SARS-CoV-2 IgG. Only 1 respondent reported a case of transmission after clearing precautions based upon 2 tests with Ct >35.

**Table 1.** Respondent Opinion of COVID-19 Recovery among Asymptomatic Case Scenarios Using Solo Criterion to Discontinue Precautions

Solo Criterion			
Asymptomatic Case Scenario	Believed Case Was Recovered and Not Infectious, No. (%)	Used Criterion to Clear Precautions, No. (%)^a^	Transmission Events Among Those Released from Precautions Using This Criterion, No. (%)^b^
**History of COVID-like symptoms** Clear history of COVID-like symptoms 1 month prior; current PCR Ct is 30; SARS-CoV-2 IgG negative	25 (42)	17 (68)	0
**Exposure to SARS-CoV-2–positive household or close contact** No history of symptoms; known SARS-CoV-2–positive household contact 5 weeks prior; current PCR Ct is 30; SARS-CoV-2 IgG negative	20 (33)	10 (50)	0
**1 high Ct** No history of symptoms or SARS-CoV-2–positive contact; current PCR Ct is >35; SARS-CoV-2 IgG negative	22 (37)	10 (45)	1
**2 high Ct results** No history of symptoms or SARS-CoV-2–positive contact; current PCR Ct is >35 on 2 separate days; SARS-CoV-2 IgG negative	32 (53)	22 (69)	1
**SARS-CoV-2 IgG positive** No history of symptoms or SARS-CoV-2–positive contact; current PCR Ct is 30; SARS-CoV-2 IgG positive	33 (55)	17 (52)	0

Note. Ct, cycle threshold; IgG, immunoglobulin G to SARS-CoV-2.

a Percentage calculated among those who believe the case is recovered or not infectious.

b Number of known transmission events.

Most respondents considered asymptomatic, incidentally discovered, PCR-positive cases to be recovered using any combination of the following: recent COVID-19 symptoms, recent COVID-19 household exposure, high PCR Ct or positive SARS-CoV-2 antibody (Table 2). Half of those with this opinion had used such criteria to clear precautions (45%–64%), and only 3 reported an incident of subsequent transmission. Of the 3 respondents who reported a case of COVID transmission, 1 commented that their hospital cleared precautions using a Ct lower than given in the survey (ie, Ct < 35).

**Table 2.** Respondent Opinion of COVID-19 Recovery among Asymptomatic Case Scenarios Using Dual Criteria to Discontinue Precautions

Dual Criteria			
Asymptomatic Case Scenario	Believed Case Was Recovered and Not Infectious, No. (%)	Used These Criteria to Clear Precautions, No. (%)^a^	Transmission Events Among Those Released from Precautions Using These Criteria, No. (%)^b^
**History of COVID-like symptoms and exposure to SARS-CoV-2–positive household or close contact** Patient with clear history of COVID-like symptoms 1 month prior, known SARS-CoV-2–positive household contact 5 weeks ago; current PCR Ct is 30; SARS-CoV-2 IgG negative	31 (52)	18 (58)	0
**History of COVID-like symptoms and high Ct** Patient with clear history of COVID-like symptoms 1 month prior; no SARS-CoV-2–positive contact; current PCR Ct is >35; SARS-CoV-2 IgG negative	35 (58)	21 (60)	1
**History of COVID-like symptoms and 2 high Cts** Patient with clear history of COVID-like symptoms 1 month prior; no SARS-CoV-2–positive contact; current PCR Ct is >35 on 2 separate days; SARS-CoV-2 IgG negative	39 (65)	25 (64)	0
**History of COVID-like symptoms and SARS-CoV-2 IgG+** Patient with clear history of COVID-like symptoms 1 month prior; no known SARS-CoV-2–positive contact; current PCR Ct is 30; SARS-CoV-2 IgG positive	39 (65)	22 (56)	0
**Exposure to SARS-CoV-2–positive household or close contact and high Ct** Patient asymptomatic and no history of symptoms; known SARS-CoV-2–positive household contact 5 weeks prior; current PCR Ct is >35; SARS-CoV-2 IgG negative	30 (50)	13 (43)	0
**Exposure to SARS-CoV-2–positive household or close contact and 2 high Ct values** No history of symptoms; known SARS-CoV-2–positive household contact 5 weeks prior; current PCR Ct is >35 on 2 separate days; SARS-CoV-2 IgG negative	36 (60)	18 (50)	1
**Exposure to SARS-CoV-2–positive household or close contact and SARS-CoV-2 IgG+** No history of symptoms; known SARS-CoV-2–positive household contact 5 weeks ago; current PCR Ct is 30; SARS-CoV-2 IgG positive	37 (62)	17 (46)	0
**High Ct and SARS-CoV-2 IgG+** No history of symptoms; no known SARS-CoV-2–positive contact; current PCR Ct is >35; SARS-CoV-2 IgG positive	38 (63)	19 (50)	1
**2 high Ct values and SARS-CoV-2 IgG+** No history of symptoms; no known SARS-CoV-2–positive contact; current COVID PCR Ct is ≥35 on 2 separate days; SARS-CoV-2 IgG positive	50 (83)	24 (48)	1

Note. Ct, cycle threshold; IgG, immunoglobulin G to SARS-CoV-2.

a Percentage calculated among those who believe the case is recovered or not infectious.

b Number of respondents (n = 3) reporting known transmission events when using 4 of the dual criteria.

In conclusion, early in the COVID-19 pandemic, leaders of infection prevention programs were driven to develop local protocols to distinguish infectious COVID-19 cases from convalescent cases to avoid harm due to delayed admission and delayed medical care from unnecessary isolation precautions. This survey of real-time hospital experiences showed that these efforts to clear unnecessary isolation were common and used simple criteria based on recent COVID-19 symptoms, recent COVID-19 household exposure, high PCR Ct, or positive SARS-CoV-2 antibody. It also suggests that decisions by infection prevention experts to clear precautions may be safely performed with minimal resultant COVID-19 transmission.

This experience raises the importance of providing early guidance in a pandemic that mitigates isolation harm from widespread screening with overly sensitive tests. The widespread use of routine PCR screening in healthcare coupled by the tendency for PCR tests to remain positive for at least 3 months after COVID-19 resulted in the flagging of numerous noninfectious convalescent cases for single rooms and COVID-19 precautions. The likelihood of harm from unnecessary precautions that impeded or delayed care increased after every COVID-19 wave due to the increased prevalence of convalescent cases in the community. These unintended consequences are why recent national guidance has urged the discontinuation of routine pre-procedural and admission testing for COVID-19 in the setting of widely available effective vaccines, high vaccine uptake among healthcare providers, and the circulation of less virulent SARS-CoV-2 strains.

This study had several limitations. The survey was conducted when SARS-CoV-2 PCR and antibody testing were just emerging and effective vaccines were early in their distribution. Responses are therefore likely conservative. Since then, use of antibody testing has declined and greater experience with Ct has resulted in increasing clearance from COVID-19 precautions at lower Ct values (eg, 30).^10^ Second, the survey addresses general circumstances and does not address immunocompromised hosts, which may have prolonged contagiousness and are best managed on a case-by-case basis. This survey is subject to bias related to volunteerism and recall. Respondents also disproportionally reflect larger academic institutions which were more likely to have access to and experience with Ct.

In a convenience sample of hospital infection prevention experts, simple criteria were often used to distinguish recently recovered COVID-19 cases from those who were currently infectious when asymptomatic patients were routinely screened by SARS-CoV-2 PCR and were incidentally positive. Use of highly sensitive molecular tests that remain positive long after infectiousness has passed raises the need to provide guidance early in a pandemic to prevent harm from unnecessary precautions that delay admission and impede medical care.

## Supplementary material

For supplementary material accompanying this paper visit https://doi.org/10.1017/ice.2023.147. click here to view supplementary material
