# Supplementary material for: Assessing past versus present severe acute respiratory coronavirus virus 2 (SARS-CoV-2) infection: A survey of criteria for discontinuing precautions in asymptomatic patients testing positive on admission
Source: Infect Control Hosp Epidemiol. 2023 Sep 13;45(2):237–40. doi: 10.1017/ice.2023.147 (PMC10877527; doi:10.1017/ice.2023.147)
Supplement: Supplementary file 1 [file S0899823X23001472sup001.docx]

**Survey of Criteria for Recovered COVID-19 Cases**

**Why is This Survey Needed?** COVID-19 patients can remain positive by PCR-testing for several months. Pre-admission or pre-procedure testing can identify recovered asymptomatic patients who may no longer be contagious but who would require COVID precautions according to current national recommendations (10 days). This can result in unintended consequences including delays in appropriate care. Examples include:

- A psychiatric patient unable to relay symptoms is required to quarantine for 10 days before transfer to inpatient psychiatric ward for needed therapy
- A pregnant patient is found to be asymptomatic but COVID positive and has to use precautions with her newborn for 10 days
- A patient with terminal cancer pending palliative chemotherapy is found to be COVID-positive prior to port-a-cath placement and the procedure is rescheduled

**Objectives:** (1) To assess what criteria are being applied to identify recovered COVID cases. (2) To assess whether these criteria are being used to discontinue COVID precautions.

**Survey Directions:** This survey evaluates your opinion about what criteria help identify recovered COVID-19 cases. There are no right or wrong answers. Responses will be aggregated across facilities. Individual responses will not be published. You can choose to stop at any time. Please see attached Study Information Sheet for more information.

**Intended Survey Respondent:** Infection prevention leadership, or team member.

**The survey takes approximately 10-15 minutes to complete. You may download the survey to preview.**

**This survey allows for saving your partial answers and returning at a later time to complete it.**

**SRN ID (If Applicable):___________**

**Date of Survey Completion: ______________**

**Facility name not needed. A de-identified code name for your facility will be assigned upon survey receipt.**

| Facility Characteristics | | | | | | | | | | | | | | | |
| --- | --- | --- | --- | --- | --- | --- | --- | --- | --- | --- | --- | --- | --- | --- | --- |
| Location (State) |  | | | | | | | | | | | | | | |
| Number of acute care beds | <200 | | | | 200-399 | | | | | 400-599 | | | 600+ | | |
| Type of facility  (Check all that Apply) | Community Hospital  Academic Medical Center  Tertiary/Quaternary referral center  Oncology  Level 1 or 2 Trauma Center  HSCT/BMT/Transplant Center  Dedicated Specialty Hospital (only admits specific patient populations):  Dedicated Orthopedics  Dedicated Pediatrics  Dedicated Psychiatric  Dedicated Long-Term Acute Care | | | | | | | | | | | | | | |
| Total number of hospitalized COVID patients to date | < 200 | | | 200 - 399 | | | | | 400 - 599 | | | 600+ | | | |
| Respondent Characteristics: | | | | | | | | | | | | | | | |
| Infection Prevention Position  (Check all that Apply) | Hospital Epidemiologist/Medical Director  Infection Prevention Manager/Director  Infection Preventionist  Chair of Infection Prevention and Control Committee  Other (Please Specify): __________________________ | | | | | | | | | | | | | | |
| Degree  (Check all that Apply) | RN | MD | | | | Masters | | CIC (Certification in Infection Control) | | | | | | Other (Please Specify):  __________________ | |
| Years in Infection Prevention | <2 years | | 2 -< 5 years | | | | 5 -< 10 years | | | | 10+ years | | | | N/A |
| Years in Infectious Diseases  (For Physician Respondents) | <2 years | | 2 -< 5 years | | | | 5 -< 10 years | | | | 10+ years | | | | N/A |

This survey will evaluate 5 possible criteria for determining recovered COVID patients. For each of the below scenarios, please assume the patient is a typical host (**NOT immunocompromised**), has had **no prior positive COVID test**, and is **currently asymptomatic** for COVID-related symptoms (e.g. admitted for reasons unrelated to COVID).

Please review the below criteria for discontinuation of precautions. You will be asked questions about a series of scenarios where solo criterion are present, followed by varying combinations of dual criteria for whether you would consider a patient recovered.

The following five criteria will appear in the scenarios:

***A. HISTORY OF COVID-LIKE SYMPTOMS***

Clear history of COVID-like symptoms (at minimum ≥ 10 days ago, can be distant)

***B. EXPOSURE TO COVID+ HOUSEHOLD OR CLOSE CONTACT***

Clear exposure to confirmed COVID (e.g. household or close contact) ≥ 24 days ago (14 days incubation + 10 days contagious period)

***C. HIGH CYCLE THRESHOLD***

Single COVID PCR cycle threshold is high (≥ 35)*

***D. TWO HIGH CYCLE THRESHOLDS***

COVID PCR cycle threshold high (≥ 35)* in both tests taken at least a day apart

***E. SEROLOGY—COVID IgG+***

Positive COVID IgG

*Given the many different assays available, for the purposes of this survey, please assume that for COVID-19 PCR, a cycle threshold (CT) of 35 is considered at or above the upper limit of detection.

| Example Scenario | Do you believe the case is recovered (not infectious)? | If yes, # cases you have cleared using these criteria? (if no, leave blank) | If you have cleared cases, how many have resulted in COVID transmission? |
| --- | --- | --- | --- |
| Survey Questions—Solo Criterion | | | |
| 1. *HISTORY OF COVID-LIKE SYMPTOMS*   Patient with clear history of COVID-like symptoms a month ago, now asymptomatic. Current COVID PCR cycle threshold is 30. No known COVID contact, COVID IgG negative. | YES  NO | 0  1-5  6-10  10+ | 0  1-5  6-10  10+ |
| 1. *EXPOSURE TO COVID+ HOUSEHOLD OR CLOSE CONTACT*   Patient asymptomatic and no history of symptoms. Known household COVID+ contact 5 weeks ago. Current COVID PCR cycle threshold is 30. COVID IgG negative. | YES  NO | 0  1-5  6-10  10+ | 0  1-5  6-10  10+ |
| 1. *HIGH CYCLE THRESHOLD*   Patient asymptomatic and no history of symptoms. No known COVID+ contact. Current COVID PCR cycle threshold is >35. COVID IgG negative. | YES  NO | 0  1-5  6-10  10+ | 0  1-5  6-10  10+ |
| 1. *TWO HIGH CYCLE THRESHOLDS*   Patient asymptomatic and no history of symptoms. No known COVID+ contact. Current COVID PCR cycle threshold is >35 on two separate days. COVID IgG negative. | YES  NO | 0  1-5  6-10  10+ | 0  1-5  6-10  10+ |
| 1. *COVID IgG+*   Patient asymptomatic and no history of symptoms. No known COVID+ contact. Current COVID PCR cycle threshold is 30. COVID IgG positive. | YES  NO | 0  1-5  6-10  10+ | 0  1-5  6-10  10+ |
| Survey Questions – Dual Criteria | | | |
| 1. *HISTORY OF COVID-LIKE SYMPTOMS AND EXPOSURE TO COVID+ HOUSEHOLD OR CLOSE CONTACT*   Patient with clear history of COVID-like symptoms a month ago, now asymptomatic. Had known household COVID+ contact 5 weeks ago. Current COVID PCR cycle threshold is 30. COVID IgG negative. | YES  NO | 0  1-5  6-10  10+ | 0  1-5  6-10  10+ |
| 1. *HISTORY OF COVID-LIKE SYMPTOMS AND HIGH CYCLE THRESHOLD*   Patient with clear history of COVID-like symptoms a month ago, now asymptomatic. No known COVID+ contact. Current COVID PCR cycle threshold is >35. COVID IgG negative. | YES  NO | 0  1-5  6-10  10+ | 0  1-5  6-10  10+ |
| 1. *HISTORY OF COVID-LIKE SYMPTOMS AND TWO HIGH CYCLE THRESHOLDS*   Patient with clear history of COVID-like symptoms a month ago, now asymptomatic. No known COVID+ contact. Current COVID PCR cycle threshold is >35 on two separate days. COVID IgG negative. | YES  NO | 0  1-5  6-10  10+ | 0  1-5  6-10  10+ |
| 1. *HISTORY OF COVID-LIKE SYMPTOMS AND COVID IgG+*   Patient with clear history of COVID-like symptoms a month ago, now asymptomatic. No known COVID+ contact. Current COVID PCR cycle threshold is 30. COVID IgG positive. | YES  NO | 0  1-5  6-10  10+ | 0  1-5  6-10  10+ |
| 1. *EXPOSURE TO COVID+ HOUSEHOLD OR CLOSE CONTACT AND HIGH CYCLE THRESHOLD*   Patient asymptomatic and no history of symptoms. Known household COVID+ contact 5 weeks ago, current COVID PCR cycle threshold is >35. COVID IgG negative. | YES  NO | 0  1-5  6-10  10+ | 0  1-5  6-10  10+ |
| 1. *EXPOSURE TO COVID+ HOUSEHOLD OR CLOSE CONTACT AND TWO HIGH CYCLE THRESHOLDS*   Patient asymptomatic and no history of symptoms. Known household COVID+ contact 5 weeks ago. Current COVID PCR cycle threshold is >35 on two separate days. COVID IgG negative. | YES  NO | 0  1-5  6-10  10+ | 0  1-5  6-10  10+ |
| 1. *EXPOSURE TO COVID+ HOUSEHOLD OR CLOSE CONTACT AND COVID IgG+*   Patient asymptomatic and no history of symptoms. Known household COVID+ contact 5 weeks ago, current COVID PCR cycle threshold is 30. COVID IgG positive. | YES  NO | 0  1-5  6-10  10+ | 0  1-5  6-10  10+ |
| 1. *HIGH CYCLE THRESHOLD AND COVID IgG+*   Patient asymptomatic and no history of symptoms. No known COVID+ contact. Current COVID PCR cycle threshold is >35, and COVID IgG positive. | YES  NO | 0  1-5  6-10  10+ | 0  1-5  6-10  10+ |
| 1. *TWO HIGH CYCLE THRESHOLDS AND COVID IgG+*   Patient asymptomatic and no history of symptoms. No known COVID+ contact. Current COVID PCR cycle threshold is ≥ 35 on two separate days. COVID IgG positive. | YES  NO | 0  1-5  6-10  10+ | 0  1-5  6-10  10+ |

Please provide any additional comments below:

***Thank you for completing the survey!***
